# Exosomal miRNAs as biomarkers for diagnostic and prognostic in lung cancer

**DOI:** 10.1002/cam4.3379

**Published:** 2020-08-10

**Authors:** Jing Wu, Zuojun Shen

**Affiliations:** ^1^ Department of Clinical Laboratory Anhui Provincial Hospital Affiliated to Anhui Medical University Hefei Anhui P.R. China; ^2^ Department of Clinical Laboratory Division of Life Sciences and Medicine The First Affliated Hospital of USTC University of Science and Technology of China Hefei Anhui P.R. China

**Keywords:** biomarker, diagnostic, exosomal miRNA, lung cancer, non‐coding RNA, prognostic

## Abstract

More and more studies report that exosomes released by various cells can serve as a medium for information exchange between different cells. Through a deep understanding of the physical and chemical properties of exosomes, the researchers revealed a more precise molecular mechanism of its participation in the process of intercellular communication. In particular, microRNA (miRNA) is found inside exosomes, as well as long noncoding RNA (lncRNA). Extensive evidence indicates that exosomal miRNAs participates in the occurrence and development of lung cancer and plays a variety of roles. Therefore, the release of RNA‐containing exosomes in many different kinds of body fluids has caused widespread interest among researchers. In this review, we report evidence from human studies involving miRNAs and other ncRNAs in exosomes associated with lung cancer as diagnostic and prognostic markers. Currently, there is a small amount of evidence that exosomal miRNAs can be used as early diagnosis and prognostic markers for lung cancer, and their exact role in lung cancer patients still needs further study.

## Graphical abstract

In this review, we report evidence from human studies involving miRNAs and other ncRNAs in exosomes associated with lung cancer as diagnostic and prognostic markers. Currently, there is a small amount of evidence that exosomal miRNAs can be used as early diagnostic and prognostic markers for lung cancer, and their exact role in lung cancer patients still need further study.

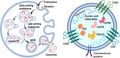

## 1 INTRODUCTION

According to the 2018 global cancer statistics, lung cancer is the cancer with the highest morbidity (1.6 percent of total cases) and mortality (18.4 percent of total cancer deaths) in the world.^1^ Unfortunately, despite the decline in lung cancer mortality, the majority of patients are still diagnosed with advanced or metastatic lung cancer, leading to poor outcomes.^2^ Therefore, detection of lung cancer in the early stage before clinical symptoms, which may be an effective means to reduce cancer mortality. So it is worth our efforts to find effective and reliable biomarkers. Despite advances in the treatment of lung cancer, the prognosis of patients is not satisfactory.^3^ At present, we urgently need to find biomarkers that may predict the recurrence of lung cancer after surgery to improve the prognosis of patients.

**Figure 1:**
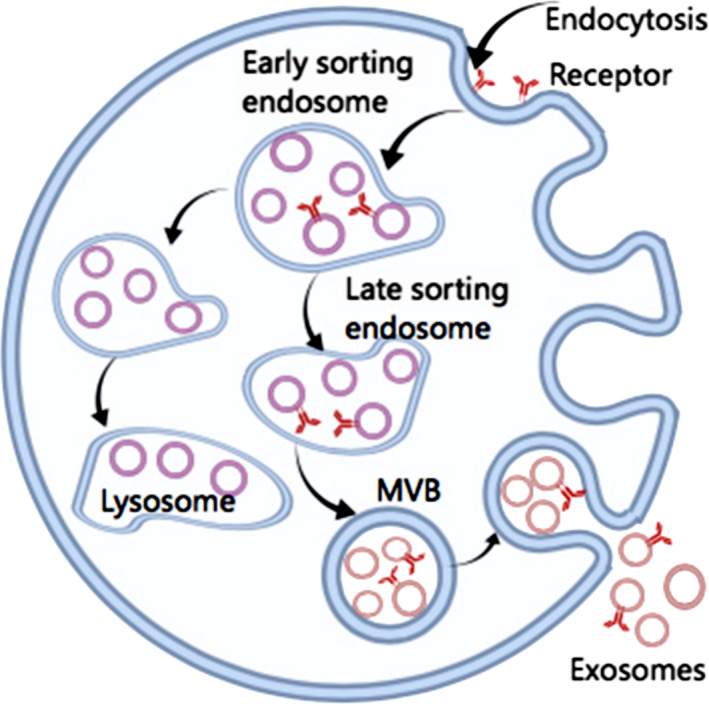
Exosomes sprout to form early multivesicular bodies (MVB). When fused with the plasma membrane, they form intracellular vesicles (ILV) and are released into the extracelluar environment

Exosomes are vesicles with a diameter of 40‐100 nm. They sprout to form early multivesicular bodies (MVB). When fused with the plasma membrane, they form an intracellular vesicle (ILV), which is released into the extracellular environment (Figure 1).^4^, ^5^ It is noteworthy that most cells can secrete exosomes. Therefore, exosomes are widely present in a variety of biological fluids, such as semen,^6^ urine,^7^ serum,^8^ plasma,^9^ saliva,^10^ bile,^11^ breast milk,^12^ amniotic fluid,^13^ cerebrospinal fluid,^14^ ascites,^15^ etc. Exosomes can be separated by ultracentrifugation,^16^ precipitation,^17^, ^18^ and microfluidic chip.^19^, ^20^, ^21^ Exosomes can be extracted from human body fluids by using different separation methods, and the number of exosomes in serum and plasma samples is more consistent ,^22^ the number of plasma exosomes is greater than the number of bronchoalveolar lavage (BAL) exosomes.^23^ Compared with serum samples and BAL samples, plasma exosomes extracted by ultracentrifugation contain more biomarkers.^23^, ^24^ Currently, there are many methods to detect the composition of exosomes, such as real‐time PCR，enzyme‐linked immunosorbent assay (ELISA), flow cytometry analysis and Western Blot (WB).^25^, ^26^ More and more evidence shows that the increase and dysregulation of exosomes secretion in cancer cells are related to tumorigenesis,^27^ and exosomal miRNAs biomarkers play an important role in many cancers, such as nasopharyngeal carcinoma, lung cancer, and colorectal cancer.^28^ Exosome is also expected to be a liquid biomarker for the diagnosis, prognosis, and treatment of head and neck squamous cell carcinoma ,^29^ but it has not yet been found that it can be used to connect barrett's esophagus and esophageal adenocarcinoma patients with other subjects distinguishing circulating exosomal miRNAs.^30^ The latest evidence shows that exosomes are closely related to the occurrence of lung cancer, and that tumor‐derived exosomes can be involved in the occurrence and development of lung cancer by regulating multiple pathways, such as enhancing tumor angiogenesis and vascular permeability,^31^ participating in epithelial‐mesenchymal transformation (EMT)^32^ and promoting chemotherapy resistance.^33^ These evidences show that exosomes play a crucial role in the occurrence and progression of lung cancer, and provide a new prospect for the treatment of nonsmall cell lung cancer, which needs further study.

**Figure 2:**
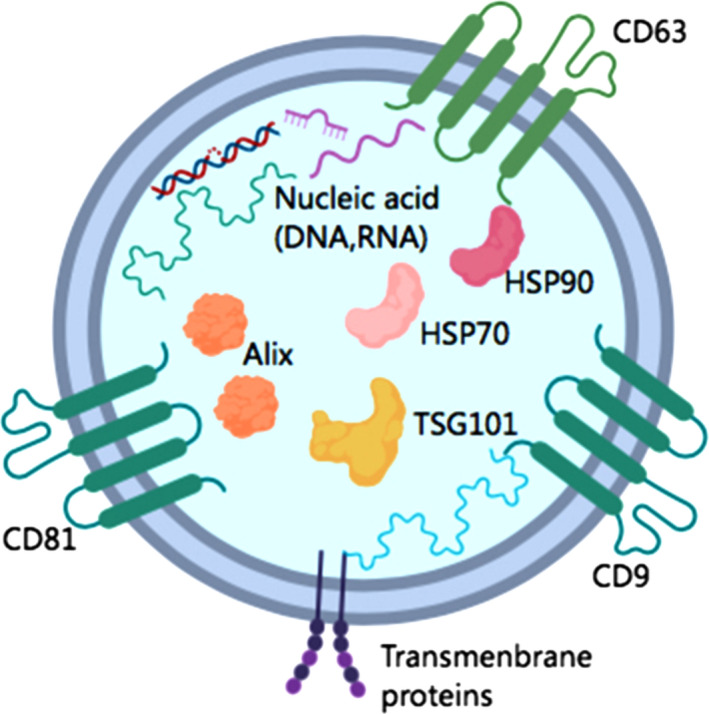
Surface markers and contents of exosomes

Recently, more and more studies have shown that exosomal miRNAs are new biomarkers of lung cancer. Exosomal miRNAs have been detected in all human body fluids and are used as noninvasive biomarkers for tumor detection.^34^ MiRNA is a small class of noncoding single‐stranded RNAs that can be silenced by combining with the corresponding 3'‐nontranslational area (3'‐UTR) or open reading frame. What we know is that RNA can be effectively packed into exosomes through a specific sorting mechanism, and this loading mechanism can occur randomly.^35^ Exosomes serve as a bridge for information exchange between cells, which can transport miRNAs and prevent RNA enzyme degradation,^36^ and can also affect cell‐cell communication by transporting their contents to target cells in the lung cancer microenvironment,^37^, ^38^ which is also the reason why exosomes can be used as ideal liquid biopsy specimens. The double‐layer lipid structure of exosomes can protect the internal miRNA from RNase degradation, with stable content, long half‐life, stably carrying various biologically active components derived from cells, and having certain cell and disease specificity. Protein markers on the surface of exosomes such as CD9 and CD63, etc can be used for the identification and screening of exosomes (Figure 2). Importantly, plasma miRNAs and plasma exosomal miRNAs can be distinguished by the treatment of exosomes extracts using a combination of protease K and RNase A, followed by analysis by agilent 2100 bioanalyzer. The results showed that exosomal RNA was slightly reduced (about 10%) by exosomes pretreatment, while RNA extracted directly from plasma was mostly reduced (about 66%).^39^ Rabinowits et al found that by comparing circulating tumor exosomes levels in plasma samples from 28 patients with lung adenocarcinoma and 9 healthy controls, 12 specific miRNAs were confirmed to be elevated in NSCLC and were reflected in circulating exosomes.^40^ The level of exosomal miRNAs in body fluids of lung cancer patients was up‐regulated, which also indicated that exosomal miRNAs played a key role in the development and progression of lung cancer. We are the first review to not only list in detail the research on exosomal miRNAs as biomarkers for the diagnosis and prognosis of NSCLC in recent years, but also list other ncRNAs in exosomes as biomarkers for lung cancer.

### 1.1 Exosomal miRNAs as diagnostic biomarkers for lung cancer

Recent studies have shown that researchers are highly concerned with exosomal miRNAs as diagnostic biomarkers for cancer detection and screening.^41^ In this regard, they focused on the function of exosomal miRNAs and their ability as biomarkers for cancer. Most of them studied the level of exosomal miRNAs in plasma, serum, or alveolar lavage fluid. In the following description, we will make a simple and powerful summary of the role of the exosomal miRNAs in different body fluids, and Table 1 summarizes all the retrieved studies.

**Table 1:** Exosomal miRNAs in different body fluids are used as diagnostic biomarkers for lung cancer

Ref	Year	Country	Fluids	n of cases and specimens	Extraction method	Regulation of miRNAs	Sensitivity and specificity for lung cancer
Cazzoli et al^42^	2013	Italy	plasma	10AC,10HC, 10granulomas (screeningphase) 50AC,25HC, 30 granulomas (validation phase）	precipitation method	742 miR‐378a↑,‐379↑ ‐139‐5P↑,‐200b‐5P↑ (divide carcinomas and healthy smokers) miR‐151a‐5p↑,‐629↑, ‐30a‐3p↑,‐200b‐5p↑ ‐154‐3p↑,‐100↑ (divide carcinomas and granulomas)	combining four miRNAs AUC=0.98 sensitivity=97.5% specificity=72.0% combining six miRNAs AUC=0.76 sensitivity=96% specificity=60%
Rodriguez et al^23^	2014	Spain	plasma BAL	30 NSCLC and 75 nontumors (screening phase) 15 mixing cases (validation phase)	ultracentrifugation	miR‐126,‐144 (specific to plasma) miR‐302a,‐302c (specific to BAL)	
Zhou et al^43^	2016	China	plasma	30AC,10HC (screening phase) 42AC,32HC (training phase) 66AC,62HC (testing phase) 33AC,30HC (validation phase)	precipitation method	39 14 6 miR‐196‐3p↑,‐21‐5P↑ ‐221‐3P↑,‐409‐3P↑ ‐425‐5P↑,‐584‐5P↑ (miR‐584‐5p expressed statistically lower level in tumor samples)	combining six miRNAs AUC=0.72, sensitivity=69%, specificity=66%; AUC=0.74, sensitivity=67%, specificity=71%; AUC=0.84, sensitivity=73%, specificity=80% for the training, the testing and validation phase respectively
Jin et al^44^	2017	China	plasma	21NSCLC,12HC (screening phase) 20NSCLC,30HC (validation phase)	ultracentrifugation	956 miR‐30a‐3p↓,‐30e‐3p↓,‐181‐5P↑,‐361‐5P↑(specific to AC) ‐15b‐5p↓,‐320b↑ ‐10b‐5p↓ (specific to AC)	combining ‐181‐5p, ‐361‐5p AUC=0.936, sensitivity=80.65% specificity=91.67% combining ‐10b‐5p， ‐320b AUC=0.911 sensitivity=83.33% specificity=90.32% combining these four miRNAs AUC=0.899 sensitivity=80.25% specificity=92.31%
Zhang et al^46^	2017	China	serum	30SCC,10HC (screening phase) 24SCC,15HC (screening phase) 44SCC,57HC (testing phase) 34SCC,36HC, 10pulmonary hamartoma (validation phase)	precipitation method	38 14 3 miR‐106a‐5P↑ miR‐20a‐5p↑ miR‐93‐5p↑	‐106a‐5p AUC=0.834 (95%CI:0.781‐0.887) ‐20a‐5p AUC=0.804 (95%CI:0.746‐0.863) ‐93‐5p AUC=0.823 (95%CI:0.767‐0.879) combining three RNAs AUC=0.832 (95%CI:0.780‐0.885)
Grimolizzi et al^49^	2017	Italy	serum	45NSCLC,31HC (validation phase)	ultracentrifugation	miR‐126↓ (in advanced NSCLC)	NSCLC vs HC AUC=0.859 (95%CI:0.737‐0.982) NSCLC‐I/II vs HC AUC=0.875 (95%CI:0.741‐1.000) NSCLC‐III/IV vs HC AUC=0.835 (95%CI:0.635‐1.000)
Feng et al^47^	2018	China	serum	23AC,16HC (validation phase)	ultracentrifugation	miR‐21‐5p↑,‐126‐3P↑, ‐140‐5p↑	miR‐21‐5p AUC=0.97 (95%CI:0.846‐0.99) miR‐126‐3P AUC=0.91 (95%CI:0.77‐0.98) miR‐140‐5P AUC=0.88 (95%CI:0.73‐0.97)
Poroyko et al^52^	2018	USA	serum	9SCLC,11NSCLC, 10HC (screening phase)	precipitation method	18 miRNAs divide NSCLC and HC 16 miRNAs divide SCLC and HC	
Shan et al^45^	2018	China	plasma	30SCC,10HC (screening phase) 32SCC,31HC (training phase) 55SCC,55HC (testing phase) 15SCC,15HC (validation phase)	precipitation method	27 12 4 miR‐181‐5P↑,‐21‐5p↑ ‐106a‐5p↑,‐93‐5p↑ (miR‐181a‐5p expressed statistically lower level in tumor samples)	‐181‐5PAUC=0.7311 (95%CI:0.661‐0.800) miR‐21‐5p AUC=0.737 (95%CI:0.670‐0.808) ‐106a‐5pAUC=0.7377 (95%CI:0.667‐0.807) ‐93‐5p AUC=0.687 (95%CI:0.614‐0.761) combining four miRNAs AUC=0.763 (95%CI:0.696‐0.829)
Kim et al^54^	2018	Korea	BAL	13AC,15HC (validation phase) 4 pairs of tissues (validation phase)	precipitation method	6 miR‐126↑	
Zhang et al^48^	2019	China	serum	43NSCLC,43HC (screening phase) 100NSCLC,90HC (training phase) 72NSCLC,47HC (validation phase)	precipitation method	6 1 ‐17‐5p↑(correlatedwith lymph node metastasis)	‐17‐5p AUC=0.746 (95%CI:0.677‐0.806) combinning ‐17‐5p and CEA,CY211,SCCA AUC=0.844 (95%CI:0.766‐0.904)
Roman‐canl et al^55^	2019	Spain	pleural lavage	21LC,25HC (screening phase) 14LC,20HC (validation phase)	ultracentrifugation	288 miR‐1‐3P↑ miR‐150‐5p↑ miR‐144‐5p↑	‐1‐3P AUC=0.914 sensitivity=92.9% specificity=95.0% ‐150‐5p AUC=0.939 sensitivity=85.7% specificity=95.0% ‐144‐5p AUC=0.925 sensitivity=78.6% specificity=95.0%
Chen et al^53^	2020	China	serum	3AC,3HC (screening phase) 62AC,62HC (validation phase)	precipitation method	60 miR‐7797↑(correlated with the N stage and TNM stage) , miR‐98‐3p↓	‐7797 AUC=0.787 (95%CI:0.705‐0.855) ‐98‐3p AUC=0.719 (95%CI:0.632‐0.796) combining two miRNAs AUC=0.816 (95%CI:0.737‐0.880)
Wu et al^50^	2020	China		48NSCLC,32LBL, 48HC (validation phase)	precipitation method	serum miR‐21‐5P↑, ‐141‐3P↑,‐222‐3p↑, ‐486‐5p↑ Exo‐miR‐146a‐5p↑, ‐486‐5p↑	combining six miRNAs AUC=0.960,P<0.0001 (95%CI:0.910‐0.987)
Sun et al^51^	2020	China	serum	72LC,72HC (validation phase)	precipitation method	miR‐106b↑(correlated with TNM stage and lymph node metastasis)	

Abbreviations: AC, adenocarcinoma; HC, healthy control; LBL, lung benign lesion; LC, lung cancer; NSCLC, nonsmall cell lung cancer; SCC, squamous cell carcinoma; SCLC, small cell lung cancer.

In earlier works in this field, Cazzoli et al analyzed the expression levels of plasma exosomal miRNAs in lung adenocarcinoma (AC) patients, pulmonary granuloma patients, and healthy smokers. They verified by PCR that exosomal microRNAs (miR‐378a, miR‐379, miR‐200b‐5p, and miR‐139‐5p) can be used to distinguish lung cancer patients from healthy people, exosomal microRNAs (miR‐151a‐5p, miR‐154‐3p, miR‐200b‐5p, miR‐629, miR‐100, and miR‐30a‐3p) can distinguish AC patients from lung granuloma patients.^42^ Since the publication of this study, this has greatly stimulated researchers' interest in the exosomal miRNAs in carcinoma. Zhou et al identified six disordered plasma exosomal miRNAs (miR‐19b‐3p, miR‐21‐5p, miR‐221‐3p, miR‐584‐5p, miR‐425‐5p, and miR‐409‐3p). These six exosomal miRNAs groups can distinguish AC patients from healthy volunteers. During the training, testing, and external verification phases, the area under the receiver operating characteristic curve (AUC) was 0.72, 0.74, and 0.84 respectively. In addition, they found that except for miR‐584‐5p, all the identified miRNAs were significantly up‐regulated in AC tissues.^43^ Other relevant evidence comes from the work of Jin et al, they found that the changes of plasma exosomal miR‐181‐5p, miR‐30a‐3p, miR‐30e‐3p, and miR‐361‐5p in AC patients were significant, while miR‐10b‐5p, miR‐15b‐5p, and miR‐320b are squamous cell carcinoma (SCC) patients specific. The authors also evaluated the accuracy of these miRNAs in classifying NSCLC, AC, and SCC, and reported the AUC as 0.899, 0.936, and 0.911 respectively.^44^ Shan et al conducted qRT‐PCR on training, testing, and external verification stages. The combination of four exosomal miRNAs (miR‐181a‐5p, miR‐21‐5p, miR‐106a‐5p, and miR‐93‐5p) can be used to detect SCC, and the AUC area of the four miRNAs groups during training, testing, and external validation stage was 0.795, 0.827, and 0.914 respectively.^45^

Parallel to the plasma samples, in many studies on humans, the expression levels of miRNAs have also been studied in serum‐derived exosomes. In the work of Zhang et al, they showed that three exosomal miRNAs combinations (miR‐106a‐5p, miR‐20a‐5p, and miR‐93‐5p) have an effective diagnostic value in male patients with SCC(AUC = 0.832). Interestingly, they reported that combinations of three miRNAs were also highly accurate in distinguishing lung SCC from lung hematoma and pointed out that the AUC value is 0.900.^46^ Similarly, in the work of Feng et al, miR‐21‐5p, miR‐126‐3p, and miR‐140‐5p showed increased expression levels in serum exosomes of AC patients compared with healthy controls.^47^ According to the research report of Zhang et al, compared with the healthy control groups, the expression of exosomal miR‐17‐5p in NSCLC patients was significantly up‐regulated. For exosomal miR‐17‐5p, the AUC value obtained by the authors was 74.6%. When the miRNA was combined with CEA, CYFRA21‐1, and SCCA, three known serological markers for the diagnosis of NSCLC, the AUC value increased to 84.4%. ^48^ One interesting result from this kind of research is that in the early stages, the serum exosomal miR‐126 levels of patients with early NSCLC and the control group were comparable, while the serum exosomal miR‐126 levels of patients with advanced NSCLC were significantly reduced. Grimolizzi et al reported that the exosomal miR‐126 could even distinguish healthy controls from patients with early NSCLC, and was more significant than the number of miR‐126 detected in serum.^49^

Recently, Wu et al reported that in early NSCLC patients, the levels of serum miRNAs (miR‐21‐5p, miR‐141‐3p, miR‐222‐3p, and miR‐486‐5p) increased significantly, as did the levels of serum exosomal miRNAs (miR‐146a‐5p and miR‐486‐5p). The combination of these six miRNAs can be beneficial to the diagnosis of early NSCLC patients, and the AUC value of this combination can be up to 0.960, the sensitivity is 85.42%, and the specificity is 92.50%.^50^ In addition, Sun et al showed that the content of serum exosomal miR‐106b in lung cancer patients was higher than that in healthy volunteers, and the level of miR‐106b was related to TNM staging and lymph node metastasis. The content of exosomal miR‐106b in the cell line is very high, and it can enhance the migration and invasion ability of lung cancer cells, and can also increase the expression of metastasis‐related proteins (MMP‐2 and MMP‐9) in the cell line.^51^

Although researchers are interested in exosomal miRNAs profiles related to the early detection of lung cancer, to our knowledge, only Poroyko et al used shotgun sequencing to study serum exosomal microRNA cargo in small cell lung cancer (SCLC), nonsmall cell lung cancer, and healthy controls. The study demonstrated that exosomal cargo is different between patients with different types of cancer and between tumor‐bearing individuals and control individuals. In cancer patients and control groups, they have identified 17 miRNAs with different expressions.^52^ Chen et al verified the highest expression of serum exosomal miR‐7797 and the lowest expression of miR‐98‐3p in patients with lung adenocarcinoma by qRT‐PCR. The diagnosis was better when the two miRNAs were combined (AUC = 0.816). They also demonstrated in vitro that increasing the expression of miR‐7797 in the A549 cell line inhibited the proliferation of lung cancer cells.^53^

The following studies were conducted in BAL. Rodriguez et al first isolated plasma and BAL exosomes from NSCLC patients and nontumor patients, and then quantified the exosomal miRNAs. Their study was the first to compare the number of exosomes in plasma and BAL. They proved that the number of plasma exosomes in both groups of patients was higher than that of BAL, and that the content of miRNAs in plasma exosomes was significantly higher than that of BAL exosomes. They also pointed out that plasma had two specific exosomal miRNAs (miR‐126 and miR‐144) and BAL had two specific exosomal miRNAs (miR‐302a and miR‐302c).^23^ Similarly, Kim et al found that the levels of the exosomal miR‐126 and let‐7a in the tumor tissues and alveolar lavage were higher in patients with lung adenocarcinoma.^54^ Other relevant evidence comes from the work of Berta et al They opened up a way to use exosomal miRNAs in pleural fluid and lavage fluid as an unexplored source of biomarkers. Specifically, lung adenocarcinoma was diagnosed specifically through the three exosomal miRNAs of miR‐1‐3p, miR‐144‐5p, and miR‐150‐5p. The authors also assessed the diagnostic capabilities of miR‐1‐3p, miR‐144‐5p, and miR‐150‐5p, and the reported AUC values were 0.914, 0.939 and 0.925 respectively.^55^

### 1.2 Exosomal miRNAs as prognostic biomarkers for lung cancer

Exosomal miRNAs test results in the blood of patients with myeloma, liver cancer, and prostate cancer have shown clinical relevance in identifying the prognosis of patients. Exosomal miRNAs profiles can also provide reliable insights into the monitoring and surveillance of lung cancer. This manuscript queries seven studies that evaluated the prognostic value of exosomal miRNAs, primarily in human tissues, plasma, and serum. In fact, in lung cancer, the use of exosomal miRNAs as clinically important biomarkers is still relatively limited in its prognosis and predictive potential, as shown in Table 2. Watabe et al found that miR‐21 significantly increased in the pleural lavage of AC patients and predicted poor disease‐free survival(DFS).^56^

**Table 2:** Exosomal miRNAs in different body fluids serve as prognostic biomarkers for lung cancer

Ref	Year	Country	Fluids	n of cases and specimens	Extraction method	Regulation of miRNAs	Prognosis	HR
Liu et al^57^	2016	China	plasma	10AC,10HC (screening phase) 196NSCLC,10HC, 11nontumor (validation phase)	precipitation method	9 miR‐23b‐3p↑ miR‐10b‐5p↑ miR‐21‐5P↑	poor OS	HR:2.42 (95%CI:1.45‐4.04) HR:2.22 (95%CI:1.18‐4.16) HR:2.12 (95%CI:1.28‐3.49)
Dejima et al^58^	2017	Japan	plasma	6NSCLC (screening phase) 195NSCLC,30HC (validation phase)	ultracentrifugation	2 miR‐21↑ miR‐4257↑	poor DFS	*P*<.05
Yuwen et al^61^	2018	China	serum	10 platinum‐resistant NSCLC,10 platinum‐ sensitive NSCLC (screening phase) 170advanced NSCLC (validation phase)	precipitation method	6 miR‐425‐3P↑	poor PFS	*P*<.0001
Liu et al^62^	2020	China	serum	105NSCLC, 60HC (validation phase)	precipitation method	miR‐216b↑	poor OS poor DFS	HR:4.06 (95%CI:1.73‐6.68) HR:4.28 (95%CI:1.82‐6.85)
Xue et al^59^	2020	China	plasma	6AC before and after surgery,6HC (screening phase) 6AC,50HC (validation phase)	ultracentrifugation	75 miR‐151a‐5P↑ miR‐10b‐5p↑ miR‐192‐5P↑ miR‐106b‐3P↑ miR‐484↑	poor OS	HR:1.44 (95%CI:1.07‐1.95) HR:1.49 (95%CI:1.08‐1.95) HR:1.40 (95%CI:0.97‐2.03) HR:1.35 (95%CI:0.98‐1.87) HR:1.29 (95%CI:0.96‐1.75)
Peng et al^60^	2020	China	plasma	5PR NSCLC,4PD NSCLC,7HC (screening phase) PR‐ pre, PD‐ pre PR‐ post,HC (validation phase)	ultracentrifugation	155 miR‐320d↑ miR‐320c↑ miR‐320b↑	poor anti‐PD1 therapy	
Watabe et al^56^	2020	Japan	pleural lavage	448AC (screening phase) 144 AC tissues, 41pleural lavage (validation phase)	precipitation method	miR‐21↑	poor DFS	*P*=.007

Abbreviations: DFS, disease‐free survival; HR, hazard ratio; OS, overall survival; PFS, progression‐free survival.

Actually, Liu et al found that plasma exosomal miR‐23b‐3p, miR‐10b‐5p, and miR‐21‐5p levels of NSCLC patients were elevated. After combining these three exosomal miRNAs with clinical variables, the AUC value increased from 0.88 to 0.91.^57^The work of DEJIMA et al found a similar finding that the levels of exosomal miR‐21 and miR‐4257 in NSCLC patients were significantly higher than those in healthy controls. They also reported that the expression levels of plasma exosomal miR‐21 and miR‐4257 in NSCLC patients who had undergone radical resection were significantly correlated with DFS.^58^ Five newly discovered plasma exosomal miR‐151a‐5p, miR‐10b‐5p, miR‐192‐5p, miR‐106b‐3p, and miR‐484 have also shown prognostic value. Xue et al found that exosomal miR‐484 increased significantly in the plasma of AC patients, but decreased significantly after surgery.^59^ Another interesting result is that compared with patients with partial remission (PR) using immunotherapy, patients with progressive disease (PD) have significantly higher exosomal has‐miR‐320d, has‐miR‐320c, and has‐miR‐320b. In addition, Zhang et al found that when the expression of the T‐cell inhibitory factor has‐miR‐125b‐5p is down‐regulated during anti‐PD‐1 treatment, patients are suitable for immunotherapy.^60^

Although there is a little information, the serum exosomal miRNAs profiles may also be effective biomarkers for monitoring the outcome of treatment. In this context, Yuwen et al found that compared with platinum‐resistant NSCLC advanced patients, the expression level of miR‐425‐3p in platinum‐sensitive patients was significantly lower, but the levels of exosomal miR‐425‐3p in both groups were still higher than healthy controls. The same phenomenon was found in cisplatin‐resistant NSCLC cell lines. In addition, they also found that the higher the exosomal miR‐425‐3p level in NSCLC patients, the worse their poor progression‐free survival (PFS).^61^ Liu et al found that the detection ability of serum exosomal miR‐216b was better than CEA, CYFRA21‐1 and SCCA, and the combination of serum exosomal miR‐216b and CEA, CYFRA21‐1 and SCCA produced an AUC value from 0.84 to 0.925. Furthermore, they found in the postoperative group that patients with miR‐216b down‐regulation (57.1%) developed lymph node metastasis.^62^

### 1.3 Other ncRNAs in exosomes: serve as biomarkers for lung cancer

In the past few years, it has been widely demonstrated that exosomes contain not only miRNAs but also long strands of noncoding RNAs (ncRNAs).^63^ More and more studies have shown that exosomes can be stable in a variety of situations and play a key role in immune response, metastasis, and drug resistance, which also provides a new therapeutic target for NSCLC treatment.^31^, ^33^, ^64^ So far, there have also been studies focusing on the analysis of ncRNAs in exosomes. As shown in Table 3, these studies mainly discussed the lncRNAs and circRNAs associated with lung cancer. Normally, the level of exosomal ncRNAs is completed by qRT‐PCR, mainly in serum.

**Table 3:** Exosomal ncRNAs in different body fluids serve as prognostic biomarkers for lung cancer

Ref	Year	Country	Fluids	n of cases and specimens	Extraction method	Regulation of ncRNAs	Clinical relevance	Sensitivity and specificity for lung cancer
Zhang et al^65^	2017	China	serum	77NSCLC,30HC	precipitation	MAlAT‐1↑	related to TNM stage and lymphatic node metastasis	AUC=0.703 sensitivity=60.1% specificity=80.9%
Teng et al^69^	2019	China	plasma	75SCC,79HC (screening phase) 10SCC,10HC 65 pairs of pre‐ and post‐ operative plasma (validation phase)	precipitation method	5lncRNA↓ 2lncRNA↑ SOX2‐OT↑	related to tumor size, TNM stage, lymphatic node metastasis	AUC=0.815 sensitivity=76.00% specificity=73.17%
Zhang et al^67^	2019	China	serum tissues	72NSCLC,64HC 27 pairs of tissues (validation phase)	precipitation method	DLX6‐AS1↑	related to disease stage, lymph node metastasis and tumor differentiation	AUC=0.806 sensitivity=77.50% specificity=885.90%
Chen et al^70^	2019	China	plasma	5AC,5HC (screening phase) 15AC,15HC (validation phase)	precipitation method	circ‐0001492↑circ‐0001346↑ circ‐0000690↑circ‐0001439↑		
He et al^71^	2020	China	plasma	21AC tissues with lymph metastasis, 20AC without; 42AC plasma with lymph node metastasis, 48AC without (validation phase)	precipitation method	has‐circR‐0056616↑	CXCR↑, related to lymph node metastasis related to T stage, M stage, TNM grade	AUC=0.812 sensitivity=79.2% specificity=90.3%
Wang et al^72^	2020	China	plasma	6SCC,6HC (screening phase) 24SCC,24HC (validation phase)	ultracentrifugation	133circRNA↑,119circRNA↓ circ‐0014235↑circ‐0025580↑ 3lncRNA↑	related to TNM stage and tumor size	AUC=0.8254 (95%CI:0.762‐0.889) AUC=0.8003 (95%CI:0.741‐0.862)
Tao et al^68^	2020	China	serum tissues	50NSCLC,50HC (training phase) 100NSCLC,100HC,10 pairs of pre‐ and post‐ operative serum (validation phase)	ultracentrifugation	TBILA↑ AGAP2‐AS1↑	TBILA related to tumor size AGAP2‐AS1related to lymph node metastasis and TNM stage	TBILA AUC=0.775 sensitivity=64.7% specificity=80.7% ‐AS1 AUC=0.734 sensitivity=66.7% specificity=73.3% combining two lncRNAs with CY211 AUC=0.853 sensitivity=91.4% specificity=80.7%
Castellao et al^73^	2020	Spain	blood	56NSCLC (validation phase)	ultracentrifugation	lncR‐p21↑	promoting angiogenesis and metastasis	TTR HR=6.129 (95%CI:1.665‐22.552) OS HR=3.745 (95%CI:1.113‐12.604)

We retrieved 4 studies on the expression of serum exosomes lncRNAs in NSCLC patients. In the study of Zhang et al, compared with healthy volunteers, exosomal MALAT‐1 was expressed at a higher level in NSCLC patients. What's more, the team of researchers demonstrated in vitro experiments that after knocking down MALAT‐1 in the NSCLC cell line, the growth and proliferation of tumor cells were inhibited, and the apoptosis of tumor cells was promoted.^65^ Similarly, Li et al found that exosomal lncRNA GAS5 was downregulated in NSCLC patients. In addition, NSCLC patients with larger tumor size and advanced TNM classification showed low levels of exosomal GAS5 expression. For exosomal GAS5, the authors obtained an AUC value of 85.7%. When the lncRNA was combined with CEA, the AUC value increased to 92.9%. It is worth noting that exosomal GAS5 can be used to distinguish patients with stage I nonsmall cell lung cancer, with an AUC value of 0.822.^66^ Zhang et al showed a significant increase in the expression level of exosomal DLX6‐AS1 in tumor tissues and NSCLC cell lines. In addition, the higher expression of DLX6‐AS1 in patients was associated with the disease stage of advanced NSCLC, positive lymph node metastasis, and poor tumor differentiation. They reported that the exosomal DLX6‐AS1 has an AUC value of 0.806, a sensitivity of 77.5%, and a specificity of 85.9%.^67^ Tao et al obtained similar findings. The levels of serum exosomal lncRNA TBILA and AGAP2‐AS1 in NSCLC patients (including AC patients and SCC patients) and early stage of NSCLC patients were higher than those in healthy controls, and also noticed that after operation, the level of these exosomal lncRNAs decreased. It is worth noting that the combination of the two exosomal lncRNAs and CFRA21‐1 showed satisfactory diagnostic results in the diagnosis of NSCLC.^68^

A new finding showed that the plasma exosomal SOX2‐OT level was significantly increased in patients with SCC. The AUC value of SOX2‐OT in the diagnosis of SCC was 0.815, and the sensitivity and specificity were up to 76% and 73.17%, respectively, indicating an effective ability. In addition, the SOX2‐OT level of exosomes was closely related to tumor size, TNM stage, and lymph node metastasis. They also noticed a significant decrease in plasma exosomal SOX2‐OT levels after SCC patients.^69^

Emerging evidence shows that exosomal circRNAs can be used as diagnostic biomarkers for cancer. So far, three studies have focused on the expression of circRNAs in plasma exosomes, two of which are on lung adenocarcinoma and one on lung squamous cell carcinoma. Chen et al Pointed out that the expression levels of has‐circ‐0001492, has‐circ‐0001346, has‐circ‐0000690, and has‐circ‐0001439 were higher in the plasma exosomes of patients with early AC, especially the highest expression of has‐circ‐0001492.^70^ In fact, according to the study of He et al, the level of exosomal hsa‐circRNA‐0056616 detected in the plasma of lung adenocarcinoma was significantly higher than that of the corresponding control. In addition, when generating a ROC curve of plasma exosomal hsa‐circRNA‐0056616 level and a diagnostic value for the diagnosis of lymph node metastasis of lung adenocarcinoma, the area under the curve is 0.812, the cut‐off value is 0.394, the sensitivity is 0.792, the specificity is 0.810, respectively.^71^ Wang et al demonstrated that increased expression of has‐circ‐0014235 and has‐circ‐0025580 in plasma exosomes of patients with lung squamous cell carcinoma. For has‐circ‐0014235 and has‐circ‐0025580, the authors obtained AUC values of 0.8254 and 8003 respectively.^72^

Despite the small amount of information, exosomes in venous blood drained by tumors can also be used as prognostic biomarkers for NSCLC. In the study by Castellano et al, they showed that the higher the level of exosomal lncRNA‐p21 in the venous blood of tumor drainage in NSCLC patients. They are associated with shorter time to relapse(TTR) and shorter overall survival(OS).^73^ In fact, in their previous studies, their results observed that poor prognosis in patients with NSCLC was associated with high levels of lincRNA‐p21 in tumor tissue. NSCLC patients with higher levels of lincRNA‐p21 will be accompanied with shorter time to relapse (TTR) and shorter overall survival time (OS).^74^ It should be pointed out that when some of the studies mentioned in the article involve AUC values, some have small sample sizes and require larger sample sizes to verify the results.

## 2 LOOKING TO THE FUTURE

Because of the lack of noninvasive and accurate detection methods, invasive detection has to adopted in clinical diagnosis which may caused harm to lung cancer patients. Therefore, it is urgent to develop noninvasive and effective detection methods to reduce the risk of death from lung cancer. At present, as far as we know, exosomal diagnostic reagents are already used in clinical trials, but they have not been used in clinical applications. Most of the hotspots of exosomes are mainly potential biomarkers, and we are also focusing on exploring the potential of exosomes in diagnosis and prognosis. We believe that in the near future, we will not only see the application of exosomes in specific diseases, but also the development of exosomes in targeted therapies. According to a detailed global survey by ISEV, differential overspeed centrifugation is the most commonly used method for exosomes separation, with density gradient centrifugation, filtration, and dimensional exclusion chromatography utilization rates of 20%, 18% and 15%, respectively.^75^ Most researchers combine the two methods to improve the efficiency and purity of exosomes.^76^ However, exosomes still have some limitations as tumor markers. First, the extraction method of exosomes. The ultra‐centrifugation can extract exosomes of large volume, but the ultra‐high speed centrifugation instrument is expensive, cumbersome, and time‐consuming.^77^, ^78^ Ultrafiltration can extract exosomes quickly without special equipment, but exosomes are easy to block the membrane or attach to the membrane and lose.^79^, ^80^ The precipitation method is easy to use without special equipment, but easy to co‐precipitate other nonexogenous pollutants.^81^ The capture technology based on immunoaffinity is very suitable for the separation of specific exosomes with high purity, high reagent cost, and low yield.^82^, ^83^, ^84^ Although sample separation by precipitation and membrane affinity is highly effective for miRNAs‐based biomarker discovery, exclusion chromatography does not distinguish patients from healthy volunteers.^85^ Recently, a method of capturing and detecting exosomes by using gold‐loaded ferric oxide nanocubes (Au‐NPFe2O3NC) was proposed. The method has low cost, simple operation, and the device is easy to carry, and can visually inspect the results, which is a highly sensitive exosome screening method.^86^ Sinna et al used exosomes membrane biomarkers CD9 and CD63 to initially isolate them, and then used tumor‐specific antibodies to quantify clinically relevant exosomes.^87^ Currently, devices based on the most popular superparamagnetic nanomaterials have the advantage of being fast and accurate and are used to detect biomarkers of low abundance biomolecules such as exosomes, but the instability, compatibility, half‐life, and susceptibility of the materials need to be considered.^88^ Oeyen et al combined ultraviolet detector and multiangle light scattering detector (AF4/ UV‐MALS) as a simple, repeatable and promising method for the characterization of urinary exosomes purity, size, and quantity.^89^ Second, the purity of the exosomes. Whether it is the most commonly used ultracentrifugation method or other extraction methods, there will be protein contamination in the extracted exosomes. The ultimate choice of separation method depends largely on the type and purity of the biological sample to be tested, the speed and cost of extraction, and downstream sequencing analysis.^90^ Third, the source of exosomes. Exosomes can be secreted by a variety of cells, such as red blood cells ^91^and macrophages.^92^ Therefore, the exosomes extracted directly cannot accurately locate whether they are secreted by normal cells or tumor cells. It seems that it is necessary to do more research on improving the yield and purity of exosomes so that exosomes can be used in clinical practice.

However, the role of miRNAs and other ncRNAs in various diseases needs to be accurately defined before we can determine whether they can be used as noninvasive biomarker. For example, exosomal miR‐21 is unregulated in lung cancer, gastric cancer, liver cancer, and other cancers.^93^, ^94^This means that its disorder cannot be uniquely associated with a particular disease and cannot be used as a specific biomarker.

It can be seen from this retrospective study that exosomal miRNAs and other ncRNAs are mainly evaluated by RT‐qPCR. The technical and biological challenges of exosomes as lung cancer biomarkers include the collection and storage of biological samples, the types of anticoagulants and the processing time of samples. Many genetic, physiological, and environmental factors related to sample heterogeneity will affect exosomes analysis.^95^ We need to pay attention to that geographical location, ethnic characteristics, and dietary habits that may influence ncRNAs expression in lung cancer patients to varying degrees. In this regard, it is worth noting that in our survey results, most of the research was conducted by Chinese people, and few studies from other countries. Therefore, it is worth spending more time to find out whether exosomal ncRNAs are more specific and sensitive than the free ncRNAs in body fluids, and whether they are more suitable as biomarkers for lung cancer.

Recent studies have shown that exosomes participate in intercellular communication and that exosomes are rich in miRNAs.^96^ Exosomal miRNAs are emerging fields in cancer research, and basic studies have shown advances in the role of exosomal miRNAs and lncRNAs in lung disease. However, only a small part of relevant studies have reported that these findings have been applied in clinical trials or clinical therapy. All in all, exosomal miRNAs are promising biomarker for lung cancer. More studies are needed to clarify the feasibility of exosomal ncRNAs in the diagnosis and prognosis of lung cancer. Further work will enable exosomal ncRNAs to be used in lung cancer patients in the near future.

## 3 AUTHOR CONTRIBUTION STATEMENT

Jing Wu screened the literature and completed the manuscript, while Zuojun Shen contributed to the selection of research direction and revised the final draft.

## CONFLICT OF INTEREST

The authors made no disclosures.

## Data Availability

No ethic approval was required as all data originated from published studies.
